# Health education needs of the Brazilian population with rheumatoid arthritis: mixed-methods cross-sectional study

**DOI:** 10.1007/s00296-026-06225-x

**Published:** 2026-07-01

**Authors:** Beatriz Cardinal Prando, Tatyane Maria Albano, Gabriel Bernardi dos Santos, Gustavo Viotto Gonçalves, Paula Regina Mendes da Silva Serrão

**Affiliations:** Department of Physical Therapy, Federal University of São Carlos, Rodovia Washington Luis, km 235 SP-310, 565-905, São Carlos, São Paulo 13.565-905 Brazil

**Keywords:** Rheumatoid arthritis, Health education, Self-management, Self-efficacy

## Abstract

Rheumatoid arthritis (RA) is a chronic, systemic inflammatory disease that impacts quality of life and functionality. Its management involves pharmacological and nonpharmacological strategies, including physical exercise and health education. Although patient education is essential, there is no consensus regarding the specific knowledge needs of individuals with RA. This study aimed to assess the health education needs of Brazilian individuals with RA using a mixed cross-sectional observational design and an online questionnaire. Adults over 18 years with a self-reported RA diagnosis were recruited via social media. The questionnaire collected sociodemographic data and information on educational needs. Quantitative data were analyzed descriptively using Microsoft Excel. A subgroup analysis explored the association between years since diagnosis and educational needs through Spearman’s correlation and simple linear regression in SPSS, after confirming non-normal distribution (p < 0.05), with a significance level of 5%. Qualitative responses were analyzed using thematic content analysis based on Bardin’s method, supported by Braun and Clarke’s inductive approach. Results indicate that individuals with RA consider it important to understand basic disease information, biopsychosocial aspects, and topics such as diet, treatment options, patient rights, pain, and physical exercise. A very weak inverse correlation was observed between years since diagnosis and educational needs (ρ = -0.160; p = 0.027), while regression analysis showed no significant predictive effect (β = -0.108; p = 0.137), with low explanatory power (R² = 0.012). The study highlights the actual educational needs of this population and identifies often-overlooked topics, providing a foundation for developing tailored educational materials.

**Supplementary Information:**

The online version contains supplementary material available at 10.1007/s00296-026-06225-x.

## Introduction

Rheumatoid arthritis (RA) is a chronic, systemic and inflammatory disease that primarily affects synovial joints 1. Due to its systemic nature, it may also manifest extra-articularly, in addition to the most common symptoms, including edema, pain, joint stiffness, and psychological disturbances. [2, 3], which results in impacts on the quality of life and functionality of individuals with RA [2, 4]. This health condition imposes a significant burden on individuals and society, primarily through psychosocial and economic impacts 5. In Brazil, there is a considerable economic impact associated with RA, driven by elevated costs resulting from direct factors, such as medical expenses, and indirect factors, such as disability retirement, absenteeism and productivity loss 6.

Recommendations for RA treatment can be categorized into pharmacological and nonpharmacological approaches 7. Therefore, a multidisciplinary team is needed to mitigate the impact of the disease on the individual 8. In this type of approach, studies show that physical exercise provides benefits, thus being recommended as part of the treatment, in addition to educating the patient and their family 8–12.

Health education is crucial for RA patients since it is a chronic disease 13. According to the *European Alliance of Associations for Rheumatology* (EULAR) recommendations, patient education must be an integral part of treatment, facilitating the individual’s understanding of necessary life adaptations concerning disease-caused changes, either physical and/or psychological 14, 15. Educational content can be delivered through disease-specific websites, healthcare apps, group education programs, individual educational counseling via booklets, and informative lectures 16. This approach is important because it enhances patients’ knowledge about their disease, improving adherence to the prescribed treatment, whether pharmacologically or non-pharmacological, and promoting self-confidence 16.

The literature indicates that RA patients have a significant need for health information, demanding it in a practical, individualized manner 17. As RA manifests differently in each individual, educational needs change on the basis of specific patient issues and demands 13. Thus, patient education must be individualized, focused on the patient, and based on their clinical condition 13, 14. Even though this evidence and recommendations advocate for patient education and although this approach has positive effects, there is still no consensus about which kind of knowledge is essential and necessary for this population.

Although essential recommendations and literature emphasize the inclusion of health education as an integral part of RA treatment 14 and given the variability of educational needs across individuals in different stages of the disease 13, leading to a lack of consensus on essential knowledge 18, studies evaluating the educational needs of the Brazilian population have not yet been conducted. Therefore, the objective of this study was to comprehensively evaluate the educational needs of this population, since there are no available resources for such evaluation through a dedicated instrument, thus helping the development of patient-centered strategies in health education for individuals with RA while considering their current needs.

## Methods

This mixed-methods cross-sectional study was conducted using an online questionnaire disseminated through social media and primary healthcare centers in the city of São Carlos, Brazil. The present study was reported in accordance with the Strengthening the Reporting of Observational Studies in Epidemiology (STROBE) Statement and adhered to the methodological and reporting recommendations for survey-based studies, considering the 16 key points proposed in the survey guidance recommendations 19.

Social media dissemination occurred through the Research Laboratory’s Instagram and Facebook pages, as well as via rheumatoid arthritis patient groups, employing a snowball sampling approach. In primary healthcare centers, dissemination was carried out through a poster displayed in the waiting room, without any involvement or mediation by healthcare professionals or staff during recruitment process. The study was approved by the Human Research Ethics Committee of the Federal University of São Carlos (n. CAAE 74583023.3.0000.5504), on January 24th, 2024. All participants provided written informed consent prior to their inclusion in the study. The study was conducted in accordance with the principles of the Declaration of Helsinki (October 2024 revision).

The study was conducted between March and September 2024. The inclusion criteria were having an RA diagnosis, being over 18 years of age, and residing in Brazil. The exclusion criteria were disagreement to participate in the research, absence of an RA diagnosis, and not residing in Brazil at the time of evaluation. The diagnosis of RA was self-reported by the participants.

All participants were invited to complete an online questionnaire on the Google Forms platform, which is available via link. Prior to questionnaire dissemination and data collection, the questionnaire was revised, and a pilot study was conducted with healthcare students and/or healthcare professionals (*n* = 5) and lay individuals (*n* = 6) to identify potential barriers or difficulties in comprehending questions intended for participants.

The online questionnaire initially presented the informed consent form (ICF), the reading and consent of which were required to proceed to subsequent sections, namely, personal and sociodemographic data and questions regarding educational needs. Participants rated each item according to their preferences, using a 5-point Likert scale ranging from “not important” to “extremely important”. This section was developed based on the Educational Needs Assessment Tool (ENAT) 20 scale, given that this resource, although validated for use with individuals with RA, has not yet been validated or translated to Brazilian Portuguese. Furthermore, an optional open-ended question was included, which stated, “Use this space to describe other information that you believe is important to know and that has not been mentioned above about your disease (rheumatoid arthritis)”. In the final section, the participants were thanked, and the researcher’s phone number and email were provided for questions and suggestions, in addition to a booklet with information about the disease, which was available for download and produced by the researchers.

All questionnaire items were mandatory, so there were no incomplete data at the end of the research. For participants who did not agree to participate, only the first and last sections were available.

All the data obtained in this study were confidential, automatically collected, and stored via Google Forms, with access restricted to the researchers. Confidentiality was maintained regarding participant involvement, and participants could request access to the results upon completion of the study.

### Statistical analysis

The quantitative data were tabulated in a Microsoft Excel spreadsheet (2013 version, Microsoft, Inc., Redmond, WA), and a descriptive analysis was performed by calculating the absolute frequency, relative frequency, mean and standard deviation.

The qualitative data of the open-ended questions were analyzed via Bardin’s thematic content analysis 21 and Braun and Clarke’s inductive thematic analysis 22. The extraction and verification of answers were performed by three researchers to ensure accuracy. Following repeated reading for familiarization, codes and themes were manually generated inductively and adjusted by consensus without the use of specific software. Validation and reliability were ensured through an audit by an external member with experience in qualitative studies. The data were managed via Microsoft Word^®^ and Excel^®^, and the results are presented, identifying participants by the order in which they completed the form (P(n), where n represents the order number).

In addition, a subgroup analysis was conducted to further investigate the data on years since diagnosis and educational needs within the sample using IBM SPSS Statistics 26. The educational needs score was calculated as the mean of the responses to 27 items rated on a 0–5 Likert scale, where higher scores indicated greater educational needs. The normality of the variables was verified using the Kolmogorov-Smirnov and Shapiro-Wilk tests. As both variables showed non-normal distributions (*p* < 0.05), nonparametric analyses were performed.

The association between years since diagnosis and educational need was investigated using Spearman’s correlation test. Subsequently, simple linear regression was performed to evaluate whether years since diagnosis predicted educational needs. In the regression, educational needs were considered the dependent variable and years since diagnosis the independent variable. For all analyses, a significance level of 5% (*p* < 0.05) was adopted.

## Results

Following questionnaire dissemination, 200 responses were received. Of these, 197 individuals consented to participate, whereas 3 declined participations. Among consenting participants, 2 individuals without a diagnosis of rheumatoid arthritis and 2 non-residents of Brazil were excluded. Additionally, 1 duplicate response was identified and removed.

The final sample comprised 192 individuals of both sexes with a mean time of diagnosis of 8.62 years (± 8.23), a mean age of 47.4 years (± 10.49), predominantly females (96.4%), who had completed higher education (41.7%) and were professionally active (54.68%). There were participants from the five regions of Brazil, the state of São Paulo being the one that encompassed most of the participation in the research, with 92 valid answers.

Regarding the type of treatment currently received, 58.85% of participants reported receiving exclusively pharmacological treatment, while 33.85% reported receiving a combination of pharmacological and non-pharmacological treatment. In contrast, 3.12% reported receiving only non-pharmacological treatment, and 4.16% did not receiving any type of treatment at the time of data collection. Sociodemographic data and characteristics of participants are presented in Table 1.

**Table 1:** Sociodemographic characteristics of participants

Characteristics	Total	*N*
Age (years), mean ± SD	47.4 ± 10.49	192
Sex		
Females Male	185 (96.4%) 7 (3.6%)	192 192
Time of diagnosis (years), mean ± SD	8.62 ± 8.23	192
Educacional level Illiterate Incomplete elementary education Complete elementary education Incomplete high school education Complete high school education Incomplete higher education Complete higher education	0 (0%) 7 (3.6%) 7 (3.6%) 13 (6.8%) 60 (31.3%) 25 (13%) 80 (41.7%)	192 192 192 192 192 192 192
Occupation		
Unemployed	27 (14.06%)	192
Housewife	26 (13.54%)	192
Retired	27 (14.06%)	192
Receiving sickness benefits/on medical leave	7 (3.65%)	192
Employed	105 (54.68%)	192
Region North Northeast Midwest Southeast South	11 (5.73%) 7 (3.65%) 7 (3.65%) 139 (72.39%) 28 (14.58%)	192 192 192 192 192
Treatments		
Pharmacological treatment only	113 (58,85%)	192
Non-pharmacological treatment only	6 (3,12%)	192
Pharmacological + non-pharmacological	65 (33,85%)	192
No treatment	8 (4,16%)	192

**Fig. 1:**
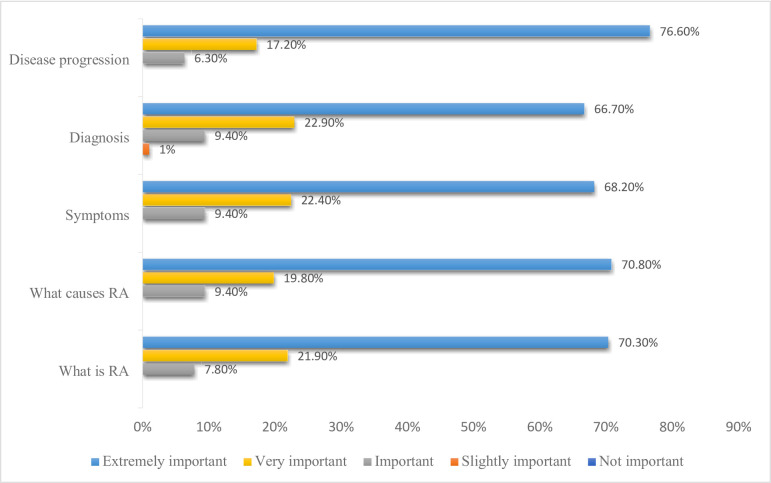
Questions regarding disease information

**Fig. 2:**
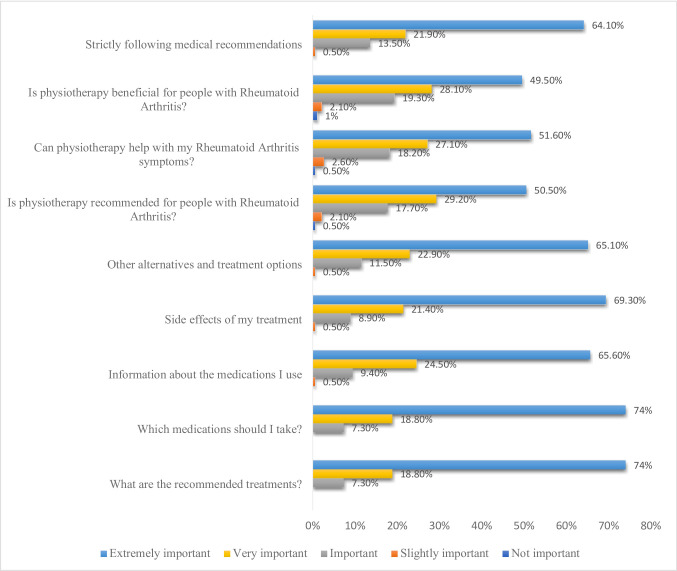
Questions regarding treatments

**Fig. 3:**
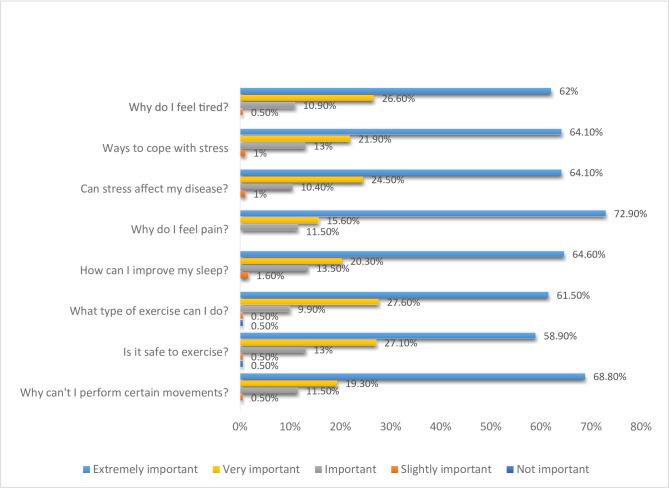
Questions regarding physical exercise, pain and biopsychosocial factors

The following questions regarding educational needs were categorized for clarity and data visualization: disease information; treatments and physical exercise; pain; and biopsychosocial factors. The response percentages for each category are illustrated in Figs. 1, 2 and 3.

The data indicate the educational needs of the Brazilian population concerning disease information, particularly regarding “disease progression”, which was rated as extremely important by 76.60% of the participants. Other high-priority themes included “what causes RA” (70.8%), “what is RA” (70.3%), “symptoms” (63.2%) and “diagnosis” (66.7%). No responses were recorded in the categories “slightly important” and “not important” for any question, indicating the relevance of all the items to this population.

Regarding treatment information, the answers that obtained the highest scores in the category “extremely important” were those regarding medication and recommended treatment, which corresponded to 74% of the answers in both. Conversely, questions regarding physical therapy treatment showed the lowest percentages, with 51.60, 50.50 and 49.50% of the responses, respectively, and were the only topics rated as “not important” in this category.

The data concerning physical exercise, detailed in Fig. 3, indicated that 68.80% of the individuals regarded exercise as “extremely important” to know why they could not execute certain movements. Additionally, 58.90 and 61.50% of the participants regarded “extremely important” issues concerning the type of physical exercise and the safety of its performance. With respect to the two questions about physical exercise, only 0.50% of the participants rated both as “not important” in this category.

For questions regarding pain, 72.90% of the participants rated “extremely important” to obtain information about “why I feel pain”. Similarly, with respect to questions regarding biopsychosocial factors, more than 60% of individuals rated themselves as “extremely important” to know about stress, fatigue and sleep.

Furthermore, 96 individuals answered the optional open-ended question about additional information regarding educational needs that they regarded as important. After review, 22 responses were excluded because they consisted of personal narratives or content not related to the study’s focus on educational needs in rheumatoid arthritis. Thematic analyses yielded the following themes: treatments, diet, legislation and rights, psychosocial aspects and disease characteristics. Themes and codes are detailed in Table 2.

**Table 2:** Themes and codes generated through content analysis

Themes	Codes (number of responses)
Treatments	Acupuncture (1)
	Use of supplements (1)
	Best treatment (1)
	Treatment with other professionals (1)
	Specific treatment location (1)
	Types of treatment (1)
	Information about treatments with low/no scientific evidence (1)
	Use of medication (2)
	Private healthcare treatment Options (1)
	Side effects of treatment (1)
	Surgery for deformities (1)
	Medical follow-up (1)
	Access to medication (4)
	Treatment in the healthcare public system (5)
	Access to quality information through brochures (1)
	Use of canabidiol (1)
Diet	Diet (7)
	Harmful foods (3)
	Benefits (5)
Legislation and rights	Laws (1)
	Disability retirement (2)
	Rights (11)
	Effects on work (1)
	Work leave (1)
	Prejudice (1)
Psychosocial aspects	Psychological conditions (2)
	Psychological support (3)
	Depression (2)
	Flares (1)
Characteristics of the disease	Adverse effects (2)
	Life expectancy (4)
	Comorbidities (1)
	Secondary diseases (6)
	Risk factors (1)
	Triggering factors (3)
	Memory (1)
	Target population (2)
	Affected body structures (2)
	Immune system function (1)
	Disease remission (3)
	Fatigue (1)
	Deformity (2)
	Cure (2)
	Specific tests (2)
	Diagnosis (2)
	Pain (3)

Regarding the “treatments” theme, 25% of the answers obtained from the open-ended question highlighted the importance of this information for this population. The participants highlighted the need for information about medication access, exemplified by reports such as P25 “*Access to medicines by SUS […]*” and P26 “*[…] the medicines provided by the healthcare unit*”. The participants also stressed the importance of information about access to the public healthcare system and available treatments, as illustrated by P53: “*Which other treatments besides allopathies*,* diet*,* exercise*,* and physical therapies do we have the right to have in public or private healthcare to improve our quality of life?”* and P25 “*Access to medication by SUS*,* treatments by the public system*,* and disability rights*”, respectively.

Some participants expressed the need to understand how diet influences the disease, particularly in terms of pain aggravation, as noted by participants in the category “diet”: P17 “*Knowing better if diet influences pain*” and P67: “*What can I eat without worsening pain*”.

Under the “legislation and rights” theme, participants sought information about disease-related benefits and rights, as indicated by P68 “*The rights concerning working conditions and benefits granted by the government*”, P33 “*The rights that concern us*,* by means of government benefits*” and P03 “*About our rightful benefits […]*”, in addition to work-related topics, such as disability retirement, leaves and effects of the disease on work.

Regarding “psychosocial aspects”, participants emphasized the influence of psychological conditions on the disease, as exemplified by P60: “*How hard my mental conditions can affect me by aggravating the disease*,* and how to overcome depression*”. They also highlighted the importance of psychological support at the beginning of the treatment, as noted by P46, “*Extremely important to know about psychological support at the beginning of the* treatment,” and P47, “*Does psychological support help to understand the disease? […]*”. Additionally, concerns about depression were expressed, as indicated by P50, “*Does depression come first or next to the RA disease?*”, and P60, “*How hard can my mental conditions affect me by aggravating the disease*,* and how to overcome depression*”.

The category “characteristics of the disease” received the most responses, underscoring its central importance. The participants sought information about secondary diseases associated with RA, as noted by P28 “*Which other diseases arise from rheumatoid arthritis?*” and P43 “*Diseases that follow RA*,* psoriasis*,* EA*,* uveitis*,* HLA b27*,* HLA b51 […]*”. The participants also expressed a desire to understand the relationship between the disease and life expectancy, as illustrated by P21: “*Whoever has this disease*,* without treatment*,* does not last for long”* and P16 “*Life expectancy and other diseases that may follow arthritis*”. Given the chronic nature of RA, participants sought information about its progression, particularly regarding pain, as noted by P13: “*Information on how to identify whether the pain that I feel is due to arthritis or something else*”; P39 reports, “*Why do we feel pain so hard on a spot and then it suddenly moves to another*”; and P12, *“The struggle to prove the chronic pain of rheumatoid arthritis*”.

Regarding the association between the number of years since RA diagnosis and educational needs, Spearman’s correlation analysis revealed a very weak inverse correlation between the number of years since diagnosis and educational needs (ρ = -0.160; *p* = 0.027), indicating that individuals who with longer disease duration tended to present slightly lower educational needs. Subsequently, simple linear regression demonstrated that years since diagnosis did not significantly predict educational needs, F(1,190) = 2.227, *p* = 0.137. The model showed low explanatory power, explaining only 1.2% of the variance in educational needs (R² = 0.012; adjusted R² = 0.006). The regression coefficient was small and non-significant (B = -0.007, SE = 0.005, standardized β = -0.108, 95% CI for B = -0.017 to 0.002), indicating that only a small proportion of the variability in educational needs can be explained by the duration of the diagnosis.

## Discussion

The present study aimed to evaluate the educational needs of the Brazilian population with rheumatoid arthritis according to their individual preferences. The study included participants from all five regions of Brazil, allowing for a broader range of responses and a greater sample diversity. Analysis of the collected data revealed varied educational needs within the population, with emphasis on fundamental disease information, as well as treatments, physical exercise, pain, and psychosocial factors. Additionally, information regarding the diet, benefits, and rights of individuals with RA was also highlighted.

Our findings reinforce the EULAR recommendation for patient education in RA [14], highlighting the importance of education being focused on individual educational needs. The recommendation notes that studies exploring patient educational needs have identified knowledge gaps in areas such as risk factors, pain management, and physical exercise, with knowledge levels ranging from low to moderate 14. This appears to be consistent with the results obtained in the study, as participants also expressed substantial education needs related to these themes, suggesting limited accessibility of such information.

A study conducted in the Netherlands revealed that the local population reported greater educational needs regarding disease processes and treatment 23, which corroborates some of the needs identified in the Brazilian population in this study. However, that study also revealed that individuals considered pain management and mobility information to be of minor importance 23, contrasting with our findings. Conversely, a study analyzing the educational needs of the European population demonstrated that such a population considers it important to know information, which aligns with our findings 24. Thus, it can be inferred that European individuals, like Brazilian individuals, recognize the relevance of information on fatigue, work-related difficulties, the benefits of physical exercise, personalized disease and treatment information, and biopsychosocial factors.

The range of educational needs, added to the relevance attributed by participants to biopsychosocial issues, such as sleep, stress, and fatigue, as well as themes such as the influence of diet on the disease, highlights the importance of health education models that go beyond basic information about the disease. These findings also reinforce the importance of a multidisciplinary team in RA management, as widely recommended in the literature to provide comprehensive patient support 25.

The need for pain-related information, particularly concerning its mechanism, was also observed. It is well established that targeted pain education, including underlying neurophysiological mechanisms, can modify pain perception and improve outcomes such as incapacity, catastrophizing, and physical performance in individuals with chronic musculoskeletal pain 26, 27. Therefore, incorporating pain neuroscience educational strategies into RA management may represent an important component of patient care.

The demand for information on laws, rights, and benefits reflects the current Brazilian context. The literature indicates that individuals with RA report an inability to perform occupational tasks compared with their pre-disease condition 5. As a result, in Brazil, people with an RA diagnosis have social security benefits 6. Although there are laws to ensure the benefits and rights of this population, a lack of information from patients can still be noticed, which may increase the social impact of the disease.

Owing to the characteristics of rheumatoid arthritis and its disabling effects, individuals tend to show a prevalence of psychological impairments, particularly depression, which may be up to twice as prevalent as in the general population 28. The findings concerning psychological issues align with the literature, given that when individuals do not receive proper treatment, their suffering impacts their emotional health and treatment adherence 28. Consequently, as highlighted by our participants, individualized treatment is crucial for understanding the psychosocial impact of RA, preventing disease progression interference, and improving management strategies.

An inverse association was found, though statistically classified as very weak, between the duration of rheumatoid arthritis and educational needs, suggesting that individuals with a longer duration of the disease tend to present slightly lower information needs. In the literature, Sierakowska et al. (2016) reported that patients with shorter disease duration presented higher Pol-ENAT scores, with a negative correlation between disease duration and total educational needs 29. However, the low explanatory power found in the regression analysis suggests that disease duration, in isolation, does not constitute a meaningful predictor of educational needs. Cruz et al. (2010) specifically reinforces the idea that disease duration, by itself, should not be interpreted as a strong determinant of educational needs, since this variable is constructed from a combination of individual, functional, emotional, and contextual factors 30.

Given the diversity of informational needs identified in this study, including disease management, treatment, self-care, rights, and psychosocial aspects, future studies focusing on developing high-quality educational resources with adequate and accessible language and thus satisfying the major needs of individuals could prove themselves relevant.

Although this study provides important insights into the educational needs of individuals with rheumatoid arthritis in Brazil, some limitations should be acknowledged. The recruitment strategy relied predominantly on digital platforms, which may have introduced selection bias by overrepresenting individuals with greater engagement in health-related information or with better digital access, potentially limiting sample representativeness. In addition, voluntary participation may have resulted in self-selection bias, as individuals with greater disease burden or unmet educational needs may have been more likely to participate, potentially overestimating educational needs. Furthermore, the proportion of participants recruited through each dissemination channel was not systematically recorded, and different recruitment methods may have reached individuals with distinct characteristics, further affecting generalizability. The sample was also predominantly composed of individuals with higher educational levels, and participants from the Southeast region of Brazil, particularly São Paulo. Finally, rheumatoid arthritis diagnosis was self-reported and not clinically confirmed. Therefore, future studies should consider multicenter designs that combine digital and paper-based recruitment strategies with active outreach methods to improve representativeness and external validity.

## Conclusion

We conclude that, based on the results obtained, the importance of health education for individuals with RA is evident, as highlighted by the qualitative findings, according to which participants consider it necessary to know about biopsychosocial aspects, diet, treatment and their own rights and benefits. Furthermore, quantitative data underscore the importance of basic disease information, physical exercise, movement performance, and pain management. Together, these findings reinforce the need for individualized and patient-centered educational strategies for individuals living with RA in Brazil. Therefore, the findings of this mixed-methods cross-sectional study may support the development of future studies and the creation of educational materials that address the specific educational needs of the Brazilian RA population, thereby clarifying key areas of interest and potentially enhancing treatment adherence.

## Supplementary Information

Below is the link to the electronic supplementary material.

Supplementary Material 1

Supplementary Material 2
