# Supplementary material for: Health education needs of the Brazilian population with rheumatoid arthritis: mixed-methods cross-sectional study
Source: Rheumatol Int. 2026 Jul 1;46(7):181. doi: 10.1007/s00296-026-06225-x (PMC13323294; doi:10.1007/s00296-026-06225-x)
Supplement: Supplementary file 1 — Supplementary Material 1 [file 296_2026_6225_MOESM1_ESM.doc]

STROBE Statement—Checklist of items that should be included in reports of ***cross-sectional studies***

|  | Item No | Recommendation |
| --- | --- | --- |
| **Title and abstract** | 1 | (*a*) Indicate the study’s design with a commonly used term in the title or the abstract  *Ok, title and line 7-8 (see Abstract document).* |
|  |  | (*b*) Provide in the abstract an informative and balanced summary of what was done and what was found  *Ok, lines 2-24 (see Abstract document).* |
| Introduction | | |
| Background/rationale | 2 | Explain the scientific background and rationale for the investigation being reported  *Ok, page 1-2, lines 2-36* |
| Objectives | 3 | State specific objectives, including any prespecified hypotheses  *Ok, page 2, lines 36-40* |
| Methods | | |
| Study design | 4 | Present key elements of study design early in the paper  *Ok, page 2, line 42* |
| Setting | 5 | Describe the setting, locations, and relevant dates, including periods of recruitment, exposure, follow-up, and data collection  *Ok, page 3, line 53* |
| Participants | 6 | (*a*) Give the eligibility criteria, and the sources and methods of selection of participants  *Ok, page 3, lines 53-57 and page 2, lines 44-48* |
| Variables | 7 | Clearly define all outcomes, exposures, predictors, potential confounders, and effect modifiers. Give diagnostic criteria, if applicable  *Ok, page 3, lines 65-69; page 5, lines 102-106 and page 3, lines 56-57* |
| Data sources/ measurement | 8* | For each variable of interest, give sources of data and details of methods of assessment (measurement). Describe comparability of assessment methods if there is more than one group  *Ok, page 3, lines 63-69* |
| Bias | 9 | Describe any efforts to address potential sources of bias  *Ok, page 3, lines 59-62* |
| Study size | 10 | Explain how the study size was arrived at  *Ok, page 5, lines 108-111* |
| Quantitative variables | 11 | Explain how quantitative variables were handled in the analyses. If applicable, describe which groupings were chosen and why  *Ok, page 4, lines 84-86* |
| Statistical methods | 12 | (*a*) Describe all statistical methods, including those used to control for confounding  *Ok, page 4, lines 84-106* |
|  |  | (*b*) Describe any methods used to examine subgroups and interactions  Ok, page 4, lines 96-106 |
|  |  | (*c*) Explain how missing data were addressed  *Not applicable* |
|  |  | (*d*) If applicable, describe analytical methods taking account of sampling strategy  *Not applicable* |
|  |  | (*e*) Describe any sensitivity analyses  *No sensitivity analyses were performed.* |
| Results | | |
| Participants | 13* | (a) Report numbers of individuals at each stage of study—eg numbers potentially eligible, examined for eligibility, confirmed eligible, included in the study, completing follow-up, and analysed  *Ok, page 5, lines 108-111* |
|  |  | (b) Give reasons for non-participation at each stage  *Ok, page 5, lines 108-111* |
|  |  | (c) Consider use of a flow diagram  *No flow diagram was used* |
| Descriptive data | 14* | (a) Give characteristics of study participants (eg demographic, clinical, social) and information on exposures and potential confounders  Ok, page 5, lines 112-122 |
|  |  | (b) Indicate number of participants with missing data for each variable of interest  *Not applicable* |
| Outcome data | 15* | Report numbers of outcome events or summary measures  *Ok, pages 6, lines 127-147 and figures 1, 2 and 3, and pages 8-9, lines 191-199* |
| Main results | 16 | (*a*) Give unadjusted estimates and, if applicable, confounder-adjusted estimates and their precision (eg, 95% confidence interval). Make clear which confounders were adjusted for and why they were included  *Not applicable* |
|  |  | (*b*) Report category boundaries when continuous variables were categorized  *Not applicable* |
|  |  | (*c*) If relevant, consider translating estimates of relative risk into absolute risk for a meaningful time period  *Not applicable* |
| Other analyses | 17 | Report other analyses done—eg analyses of subgroups and interactions, and sensitivity analyses  *Ok,* *pages 8-9, lines 191-199* |
| Discussion | | |
| Key results | 18 | Summarise key results with reference to study objectives  *Ok, page 9, lines 201-207* |
| Limitations | 19 | Discuss limitations of the study, taking into account sources of potential bias or imprecision. Discuss both direction and magnitude of any potential bias  *Ok, pages 11-12, lines 265-280* |
| Interpretation | 20 | Give a cautious overall interpretation of results considering objectives, limitations, multiplicity of analyses, results from similar studies, and other relevant evidence  *Ok, pages 9-11, lines 208-264* |
| Generalisability | 21 | Discuss the generalisability (external validity) of the study results  *Ok, pages 11-12, lines 266-277* |
| Other information | | |
| Funding | 22 | Give the source of funding and the role of the funders for the present study and, if applicable, for the original study on which the present article is based  *Ok, see tittle page* |

*Give information separately for exposed and unexposed groups.

**Note:** An Explanation and Elaboration article discusses each checklist item and gives methodological background and published examples of transparent reporting. The STROBE checklist is best used in conjunction with this article (freely available on the Web sites of PLoS Medicine at http://www.plosmedicine.org/, Annals of Internal Medicine at http://www.annals.org/, and Epidemiology at http://www.epidem.com/). Information on the STROBE Initiative is available at www.strobe-statement.org.
