# Supplementary material for: Health education needs of the Brazilian population with rheumatoid arthritis: mixed-methods cross-sectional study
Source: Rheumatol Int. 2026 Jul 1;46(7):181. doi: 10.1007/s00296-026-06225-x (PMC13323294; doi:10.1007/s00296-026-06225-x)
Supplement: Supplementary file 2 — Supplementary Material 2 [file 296_2026_6225_MOESM2_ESM.docx]

**English version of the questionnaire**

**Personal and sociodemographic data**

In this section, some personal and sociodemographic data will be collected. It is important that you answer all questions.

1. Full Name (or only initials): ______________.
2. Age: _____________.
3. Sex: (Female; Male) ____________.
4. Email: _________________________.
5. Phone number: _______________________.
6. Which city do you live? _____________________.
7. What is your weight? (in kg) __________.
8. What is your height? (e.g., 1.60 m) __________.
9. Level of education (Illiterate; Incomplete elementary education; Complete elementary education; Incomplete high school education; Complete high school education; Incomplete higher education; Complete higher education): __________.
10. Occupation (Unemployed; Housewife; Retired; Receiving sickness benefits/on medical leave; Employed): _____________________________.
11. How long have you had a diagnosis of Rheumatoid Arthritis? ________
12. What treatment are you currently receiving for the disease? (e.g., medication, physiotherapy, psychology, occupational therapy, among others) ______________.

**Questions regarding Educational Needs**

In this section, you will answer questions regarding your educational preferences and needs. Responses will be provided using a scale ranging from “Not important at all” to “Extremely important.”

For this section, please answer:

| **How important do you consider knowing about the following information:** | **Not important** | **Slightly important** | **Important** | **Very important** | **Extremely important** |
| --- | --- | --- | --- | --- | --- |
| What Rheumatoid Arthritis is? |  |  |  |  |  |
| What causes my disease? |  |  |  |  |  |
| What the main symptoms are? |  |  |  |  |  |
| How the diagnosis is made? |  |  |  |  |  |
| How my disease will progress? |  |  |  |  |  |
| Recommended treatments |  |  |  |  |  |
| Which medications I should take? |  |  |  |  |  |
| Information about the medications I use |  |  |  |  |  |
| Side effects of my treatment |  |  |  |  |  |
| Other treatment alternatives and options |  |  |  |  |  |
| Is physiotherapy recommended for people with Rheumatoid Arthritis? |  |  |  |  |  |
| Can physiotherapy help me with my Rheumatoid Arthritis symptoms? |  |  |  |  |  |
| Is physiotherapy beneficial for people with Rheumatoid Arthritis? |  |  |  |  |  |
| Strictly following medical recommendations |  |  |  |  |  |
| Why can't I perform certain movements? |  |  |  |  |  |
| Is it safe to exercise? |  |  |  |  |  |
| What type of exercise can I do? |  |  |  |  |  |
| How can I improve my sleep? |  |  |  |  |  |
| Why do I feel pain? |  |  |  |  |  |
| Can stress affect my disease? |  |  |  |  |  |
| Ways to cope with stress |  |  |  |  |  |
| Why do I feel tired? |  |  |  |  |  |

Use this space to describe any other information that you consider important to know about your disease (Rheumatoid Arthritis) that was not mentioned above: _____________________________________________________________________________________________________________________________________________________________________________________________________________________________________________________________________________________________________________________________________________________________________________.
